# Genome-Wide Expression Profiling Reveals S100B as Biomarker for Invasive Aspergillosis

**DOI:** 10.3389/fmicb.2016.00320

**Published:** 2016-03-21

**Authors:** Andreas Dix, Kristin Czakai, Jan Springer, Mirjam Fliesser, Michael Bonin, Reinhard Guthke, Anna L. Schmitt, Hermann Einsele, Jörg Linde, Jürgen Löffler

**Affiliations:** ^1^Systems Biology / Bioinformatics, Leibniz Institute for Natural Product Research and Infection Biology Hans-Knöll-InstituteJena, Germany; ^2^University Hospital Würzburg, Medical Hospital IIWürzburg, Germany; ^3^IMGM LaboratoriesMartinsried, Germany (Formerly Department of Medical Genetics and Applied Genomics, University Hospital Tübingen, Tübingen, Germany)

**Keywords:** invasive aspergillosis, allogeneic stem cell transplantation, gene expression data, fungal infection, human biomarker

## Abstract

Invasive aspergillosis (IA) is a devastating opportunistic infection and its treatment constitutes a considerable burden for the health care system. Immunocompromised patients are at an increased risk for IA, which is mainly caused by the species *Aspergillus fumigatus*. An early and reliable diagnosis is required to initiate the appropriate antifungal therapy. However, diagnostic sensitivity and accuracy still needs to be improved, which can be achieved at least partly by the definition of new biomarkers. Besides the direct detection of the pathogen by the current diagnostic methods, the analysis of the host response is a promising strategy toward this aim. Following this approach, we sought to identify new biomarkers for IA. For this purpose, we analyzed gene expression profiles of hematological patients and compared profiles of patients suffering from IA with non-IA patients. Based on microarray data, we applied a comprehensive feature selection using a random forest classifier. We identified the transcript coding for the S100 calcium-binding protein B (S100B) as a potential new biomarker for the diagnosis of IA. Considering the expression of this gene, we were able to classify samples from patients with IA with 82.3% sensitivity and 74.6% specificity. Moreover, we validated the expression of *S100B* in a real-time reverse transcription polymerase chain reaction (RT-PCR) assay and we also found a down-regulation of S100B in *A. fumigatus* stimulated DCs. An influence on the *IL1B* and *CXCL1* downstream levels was demonstrated by this S100B knockdown. In conclusion, this study covers an effective feature selection revealing a key regulator of the human immune response during IA. *S100B* may represent an additional diagnostic marker that in combination with the established techniques may improve the accuracy of IA diagnosis.

## 1. Introduction

*Aspergillus* spp. are ubiquitous molds present as saprobes in air, soil, and water. Thus, exposure to their omnipresent spores named conidia occurs constantly and the exposure to the fungus can be considerable (Oberle et al., 2015). In immunocompromised patients, deposition of conidia on mucous membranes in the lower respiratory tract may result in their germination and subsequent growth into tissue barriers. Leukemia patients, patients after allogeneic stem cell and solid organ transplantation as well as other groups of heavily immunosuppressed patients are at highest risk for *Aspergillus* infections (Leventakos et al., 2010).

*Aspergillus fumigatus* is the predominant *Aspergillus* species that causes invasive aspergillosis (IA). The rate of IA has increased 14-fold in Europe within the last two decades, with an incidence of more than 3000 patients annually in Europe (Denning, 1998). Furthermore, IA is the most expensive opportunistic infection in immunocompromised patients. *Aspergillus*-related hospitalizations cause a significant financial burden for the health care system (Slobbe et al., 2008). Specific symptoms of IA are rare and occur late in the course of infection. Thus, diagnosis of IA still remains difficult with relatively low sensitivity and specificity, despite the fact that modern assays, such as the galactomannan enzyme-linked immunosorbent assay (ELISA) and β-glucan tests as well as numerous real-time polymerase chain reaction (PCR) protocols are available. In consequence, mortality of IA is still up to 90% in central nervous system aspergillosis, but falls to 50% if patients are treated with appropriate antifungal drugs (Denning and Hope, 2010). The high clinical relevance, the rise in incidence of IA, and the *condicio sine qua non* to diagnose IA as early, specific, sensitive, and reliable as possible impose the search for new alternative biomarkers.

In this study, we therefore sought to expand the spectrum of available biomarkers for the diagnosis of IA and, to our knowledge for the first time, analyzed transcriptome profiles of hematological patients suffering from IA and compared them to profiles from hematological patients without IA and to healthy individuals. The method of analyzing the transcriptional response of the host to identify or distinguish infections has been successfully applied in other studies. Conclusions can be drawn from the transcriptomic response, since specific host reactions are caused by different conditions. For example, gene expression patterns that differentiate between active and latent tuberculosis have been determined in patients (Jacobsen et al., 2007; Lu et al., 2011). In another study, invasive candidiasis was effectively classified in mice by using a combination of different gene signatures (Zaas et al., 2010). Additionally, biomarkers for fungal and bacterial infections were discovered in human whole-blood (Dix et al., 2015). Thus, the identification of transcriptional biomarkers in the host is a promising approach.

Using a random forest-based feature selection, we found the *S100 calcium-binding protein B* (*S100B*) to be a useful specific marker. Its expression pattern allows differentiation of patients with IA from patients without IA and healthy individuals. Therefore, analysis of the *S100B* expression in patients' peripheral blood mononuclear cells (PBMCs) may contribute to an improved diagnostic sensitivity and thus facilitate a more reliable interpretation of the patients' condition. This observation was underlined by numerous accompanying functional studies, including genotyping of three single nucleotide polymorphisms (SNP) and the quantification of S100B in sera obtained from hematological patients.

## 2. Materials and methods

### 2.1. Patient characteristics

Blood samples were taken from allogeneic haematopoietic stem cell transplant (alloSCT) recipients and patients receiving myelosuppressive chemotherapy (Table 1). Clinical and microbiological data were recorded for each individual patient according to current criteria of the European Organization for Research and Treatment of Cancer/Invasive Fungal Infections Cooperative Group and the National Institute of Allergy and Infectious Diseases Mycoses Study Group (EORTC/MSG) (De Pauw et al., 2008).

**Table 1:** **Characteristics of patients and controls**.

**ID**	**EORTC classification**	**Age**	**Sex**	**Disease**	**Number of samples**
P01	Probable IA	63	f	Myeloproliferative neoplasm	2
P02	Probable IA	55	f	Multiple myeloma	3
P03	Probable IA	51	f	Acute myeloid leukemia	3
P04	Probable IA	63	f	Acute myeloid leukemia	5
P05	Probable IA	65	m	Chronic lymphocytic leukemia	2
P06	Probable IA	61	m	Myelodysplastic syndrome	2
P07	Probable IA	45	m	Acute myeloid leukemia	3
P08	Probable IA	60	m	Acute myeloid leukemia	3
P09	Unclassified	70	m	Myeloproliferative neoplasm	2
P10	Unclassified	52	m	Multiple myeloma	1
P11	Possible IFD	62	f	Acute myeloid leukemia	2
P12	Unclassified	31	m	Acute myeloid leukemia	2
P13	Unclassified	59	f	Acute myeloid leukemia	1
P14	Unclassified	61	m	Acute lymphoblastic leukemia	2
P15	Unclassified	66	f	Acute myeloid leukemia	1
P16	Unclassified	46	m	Acute lymphoblastic leukemia	2
H01	Healthy		f	–	2
H02	Healthy		m	–	1
H03	Healthy		m	–	1
H04	Healthy		f	–	1
H05	Healthy		m	–	1
H06	Healthy		f	–	1
H07	Healthy		f	–	1
H08	Healthy		f	–	1

*IA, invasive aspergillosis; IFD, invasive fungal disease; f, female; m, male*.

### 2.2. Blood sample collection

The starting point for blood sample collection in patients with probable IA and the quality of these samples for RNA extraction are crucial for this study. We initiated the collection of blood samples at the occurrence of a positive galactomannan (GM) ELISA result (defined as day 0). A positive GM ELISA result and a positive computed tomography (CT) scan are required for probable IA in the consensus definitions for invasive fungal infections, published by the EORTC/MSG (De Pauw et al., 2008). All patients with probable IA, except P05, were also PCR positive giving additional certainty for the presence of IA. Subsequently, additional whole blood (3 ml) and serum (1 ml) samples were taken, respectively. Analogous control samples were taken from patients with hematological malignancies but without any clinical signs of IA and from healthy volunteers (Table 1). Sampling was performed until patient's discharge or death (with a maximum number of samples, *n* = 5). In order to prevent RNA degradation, whole blood was drawn directly into specific collection tubes containing RNA stabilization reagent (Tempus™, Thermo Fischer Scientific, at days +3, +7, +10, +14, +18).

### 2.3. Expression data generation

RNA was extracted using the RNeasy Mini Kit (Qiagen). RNA integrity was confirmed with an Agilent 2100 Bioanalyzer (Agilent Technologies). RNA samples were hybridized to Affymetrix HG-U219 array plates. Scanned images were analyzed with AGCC 3.0 (Affymetrix) to generate CEL files (Affymetrix file format containing information about the intensity values) according to the manufacturer's instructions. The microarray data were uploaded to NCBI's Gene Expression Omnibus (Edgar et al., 2002), accession number GSE78000 (http://www.ncbi.nlm.nih.gov/geo/query/acc.cgi?acc=GSE78000).

The dataset comprises 23 samples from 8 patients suffering from probable IA with 2 to 5 samples, respectively, 13 samples from 7 unclassified patients and 1 possible invasive fungal disease (IFD) patient with 1 to 2 samples, respectively, and 9 healthy control samples from 8 donors with 1 to 2 replicates, respectively (see Table 1 for a detailed list of the patients and their characteristics).

### 2.4. Preprocessing of the gene expression data

The R package “affy” (Gautier et al., 2004) was used to read the Affymetrix CEL files as well as to perform background correction and quantile normalization according to the Robust Multi-array Average (RMA) method (Irizarry et al., 2003). In this process, a custom chip definition file (CDF) was used for probe-to-gene mapping. The CDF (version 19, “Entrez Gene”) can be downloaded at the MicroArray Lab website^1^. Afterwards, the Entrez-IDs were mapped to the corresponding gene symbols using the HGNC BioMart service^2^. The final dataset comprises 18,356 genes.

### 2.5. Identification of differentially expressed genes

Differentially expressed genes were determined using the package “limma” (Smyth, 2005) (version 3.24.14) of the programming language R. Limma analyzes the expression data by fitting linear models and determines statistical significance with moderated *t*-statistics. *P*-values were adjusted according to the false discovery rate (Benjamini and Hochberg, 1995). The genes with an adjusted *p*-value < 0.05 and at least a 2-fold up- or down-regulation were considered as differentially expressed.

### 2.6. Feature selection

**Figure 1:**
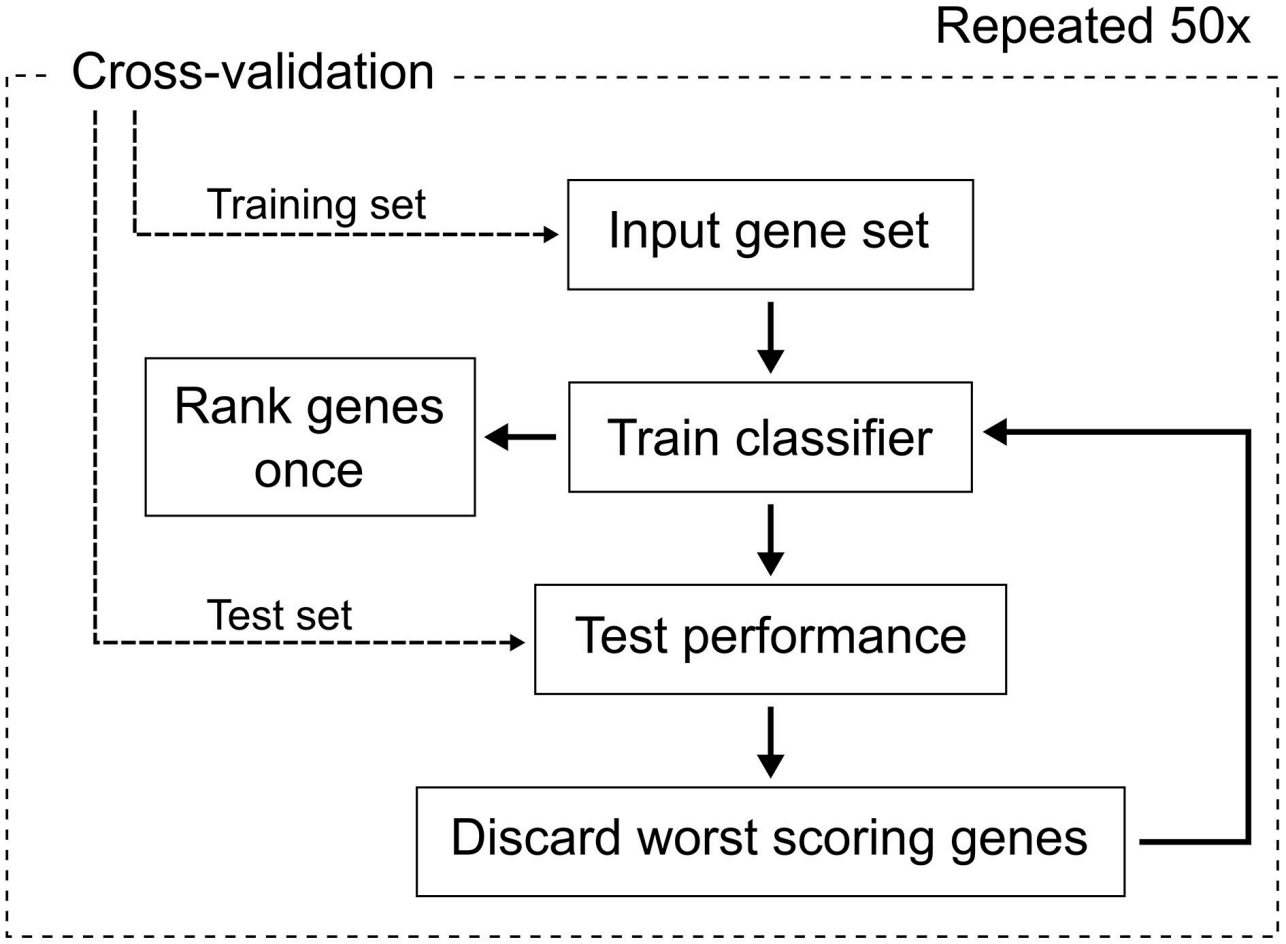
**The workflow of the feature selection process**. We used random forest as classifier and performed leave-one-out cross-validation. The ranking of the genes was done once for each fold of the cross-validation for the unreduced input gene set.

A recursive feature elimination (Guyon et al., 2002) (RFE) based on random forest (Breiman, 2001) was performed for feature selection (Figure 1). Feature selection is a technique to identify the most relevant features from a large set. In this study, the features are gene transcripts measured by microarray. Briefly, an RFE is an algorithm, which iteratively removes the worst scoring features and calculates a classification error (i.e., the proportion of wrong classifications) using the remaining features. In the presented RFE, the input data is initially classified to calculate a classification error and all features are ranked according to their importance values. The importance values were computed by random forest using the measure “mean decrease in accuracy.” Random forest calculates this measure by a permutation test, which follows the idea that a feature is more important for correct classification, the more the classification error increases, when the feature values are permuted across all samples. Therefore, it indicates how relevant a feature is for classification. Afterwards, the RFE algorithm iteratively removes the worst scoring 10% of the features and calculates the classification errors using only the remaining features. Finally, the features yielding the smallest error rate are selected. As suggested by Svetnik et al. (2004), the importance values were not recalculated in each step. To avoid a selection bias, the RFE was wrapped in a leave-one-out crossvalidation (Ambroise and McLachlan, 2002). Cross-validation is a performance assessment technique, where all samples are iteratively split into test set and training set. For leave-one-out cross-validation, the test set comprises only one sample. The test sample is used for testing our classifier. The training samples are used for feature selection and to train the classifier. The classification error is calculated only on the test sample and eventually averaged across all cross-validation iterations. Cross-validation emulates an independent test set without using additional data. The RFE with the cross-validation was repeated 50 times to control for the random effects of random forest. This whole procedure was conducted for different values of the parameters *ntree* and *mtry* of random forest. We tested 1000 and 10,000 for *ntree*. We multiplied the default value of *mtry* with the factors 0.25, 0.5, 1, 2, and 4. The default value of *mtry* is $\left\lfloor \sqrt{p}\right\rfloor$, where *p* is the number of features of the input data. The minimum error rate was calculated with the *mtry*-factor of 0.25 and *ntree* = 10,000.

### 2.7. Confirmation of selected gene expression by real-time RT-PCR assays

First strand complementary DNA (cDNA) was synthesized by using the First Strand cDNA Synthesis Kit (Thermo Fisher Scientific) according to manufacturer's instructions. Real-time PCR for 3 selected genes [*S100B, monoaminooxidase* (*MAOA*), *semaphorin 4A* (*SEMA4A*)] was performed using the iTaq Universal SYBR Green Supermix (Bio-Rad) on the StepOnePlus instrument (Thermo Fisher Scientific) according to manufacturer's instructions. Gene specific primers were: *S100B*, forward (fw) 5′-AGGGAGGGAGACAAGCACAA-3′ reverse (rv) 5′-ACTCGT GGCAGGCAGTAGTA-3′, *MAOA*, fw 5′-GCATTTCAG GACTATCTGCTGC-3′ rv 5′-TGGGTTGGTCCC ACATAAGC-3′; *SEMA4A*, fw 5′-GAGCAACAC CTCCAGTCTCC-3′ rv 5′-GGTGGTCTT TTGTGCTGCTG-3′. PCR efficiencies were calculated using the LinRegPCR program (Ruijter et al., 2009). Based on cycle of quantification (Cq) values, relative expression levels of target mRNA were calculated using the efficiency-corrected ΔΔCq method (Pfaffl, 2001) with *B2M* as the endogenous reference gene and average Cq values of probable IA patients as calibrators. Additionally, in dendritic cells (DCs) *ALAS1*, fw 5′-GGCAGCACAGATGAATCAGA-3′, rv 5′-CCTCCATCGGTT TTCACACT-3′; *CXCL1*, fw 5′-GAAAGCTTG CCTCAATCCTG-3′, rv 5′-CACCAGTGAGCT TCCTCCTC-3′; *IL1B*, fw 5′-GGACAAGCT GAGGAAGATGC-3′, rv 5′-TCGTTATCC CATGTGTCGAA-3′ were used with *ALAS1* as the reference gene.

### 2.8. Quantification of S100B levels in sera

Serum samples (50 μl each, total *n* = 75) were consecutively, longitudinally collected every 3–4 days from 8 patients after the occurrence of a positive GM ELISA assay (Platelia®, BioRad). In addition, control sera from patients without any clinical signs of IA were consecutively collected (every 3–4 days, total number of control sera *n* = 52 from 8 patients). S100B levels in sera were quantified by using the S100B sandwich ELISA kit from Abnova (Taipeh, Taiwan), according to the protocol of the manufacturer.

### 2.9. LightCycler-based melting curve analysis for genotyping

To further glean the biological role of S100B in the occurrence of IA, we screened an existing DNA archive for the presence of 3 previously described SNPs (rs9722 [*S100B*], rs2070600 [*AGER*], rs1800624 [*AGER*]) (Cunha et al., 2011). The archive contains previously collected DNA samples from allogeneic stem cell transplant recipients [33 patients with proven or probable IA and 38 controls without IA, classification according to the EORTC/MSG criteria (De Pauw et al., 2008)]. This archive allowed extending genotyping to a larger number of patients with similar risk for IA, in addition to the relatively limited number of original study patients.

Human genomic DNA was extracted by using the QIAmp Blood DNA Mini Kit (Qiagen), followed by melting curve analyses using a LightCycler®1.5 instrument (Löffler et al., 2000) and specific hybridization probes (LightSNiP, TIB MOLBIOL).

### 2.10. Generation of monocyte-derived DCs

For functional studies, DCs were generated from PBMCs as previously described (Mezger et al., 2008). Briefly, PBMCs were isolated from healthy volunteers by ficoll (Bicoll Seperation, Biochrom AG) density gradient centrifugation. Magnetic activated cell sorting with paramagnetic CD14-beads (Miltenyi Biotec) was used to further separate monocytes. Monocyte-derived DCs were generated in RPMI-1640 supplemented with 10% fetal bovine serum (Sigma Aldrich), 120 mg/l Refobacin (Merck), 10 ng/ml IL-4 (Miltenyi Biotec) and 100 ng/ml GM-CSF (Bayer Healthcare) for 5–6 days.

### 2.11. Co-culture with *A. fumigatus* and pathogen recognition receptor (PRR)-ligands

The fungal strain *A. fumigatus* ATCC 46645 (American Type Culture Collection, LGC Standards) was used for all experiments. Germ tubes were prepared as previously described (Mezger et al., 2008). Germ tubes were inactivated by incubation in 100% Ethanol for 45 min at 37°C. Co-cultivation experiments of DCs with *A. fumigatus* were performed on day 6 with a multiplicity of infection (MOI) of 1. The PRR-ligands zymosan depleted (100 g/ml) and Pam3CSK4 (100 ng/ml) (Invivogen) were used for stimulation of DCs or 6 h in the indicated concentrations.

### 2.12. RNA interference

All RNA interference experiments were performed as previously described (Mezger et al., 2008). Briefly, DCs were electroporated (EPI 2500, Dr. L. Fischer) with either short interfering double stranded *S100B*-siRNA or non-silencing, random RNA (Qiagen) at 340 V for 10 ms on day 5 after isolation and then incubated at 37°C and 5% CO_2_ for 24 h in culture medium.

## 3. Results

### 3.1. T cell regulation is specific for IA

**Figure 2:**
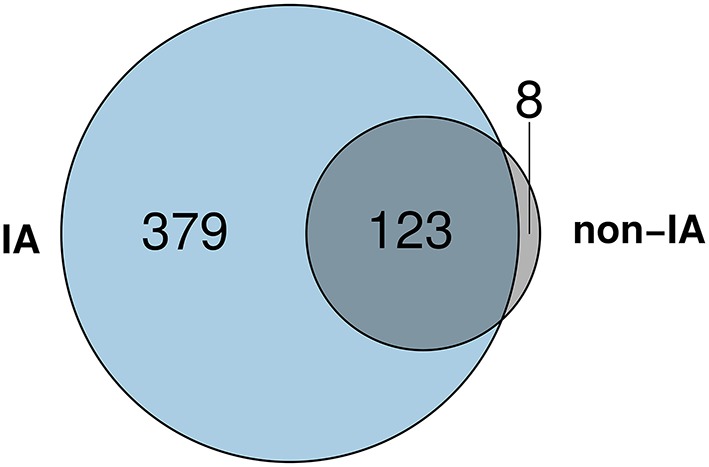
**The Venn diagram shows that 123 DEGs were identified for both IA and non-IA patients**. Additionally, 379 DEGs and 8 DEGs were specific for IA and non-IA patients, respectively.

As a first step, we identified differentially expressed genes (DEGs) in patients with IA and non-IA patients compared to healthy controls (see Section 2). Thereby, we reduced our gene set from whole-genome size to data which are associated to the underlying disease and its treatment. At a significance level of 0.05 and considering at least a two-fold change, we identified 502 DEGs for IA and 131 DEGs for non-IA samples (Figure 2). The vast majority (123 of 131) of the non-IA DEGs were also DEGs for IA. Only 8 genes were specific for the non-IA condition. We analyzed the expression patterns of these 8 genes in more detail and discovered only minimal differences between the IA and the non-IA samples (Supplementary Figure 1). Genes were expressed on a similar level and also showed similar distributions between both conditions. A direct comparison of IA and non-IA samples using the same thresholds as above yielded no significantly differentially expressed genes.

To identify gene functions significantly associated with the DEGs of patients who developed IA and patients without IA, we performed an over-representation analysis of Gene Ontology (Ashburner et al., 2000) (GO) terms. We used the tool “GOrilla” (Eden et al., 2009), which tests for enrichment by building a hypergeometric model. It adjusts the *p*-values according to the false discovery rate. The Venn diagram of the DEGs (Figure 2) contains 3 groups, which were tested for enrichment: “specific for IA,” “specific for non-IA,” “common for both conditions.” For the non-IA DEGs (*n* = 8), no significantly over-represented GO-term could be found. However, for the IA DEGs (*n* = 379) and the common DEGs (*n* = 123), GOrilla determined 161 and 39 terms, respectively, at a significance level of 0.05 (Supplementary Tables 1, 2). We found 21 terms related to T cells and/or their activation or differentiation among DEGs in IA patients. In contrast, no GO-term related to T cells was identified in the list of the common DEGs. Both lists share 23 terms, which comprise general immune responses, immune-related signaling, as well as lymphocyte and leukocyte activation and differentiation. These findings indicate a particular importance of T cells for the host response to IA.

### 3.2. *S100B* is a transcriptional biomarker for IA

**Figure 3:**
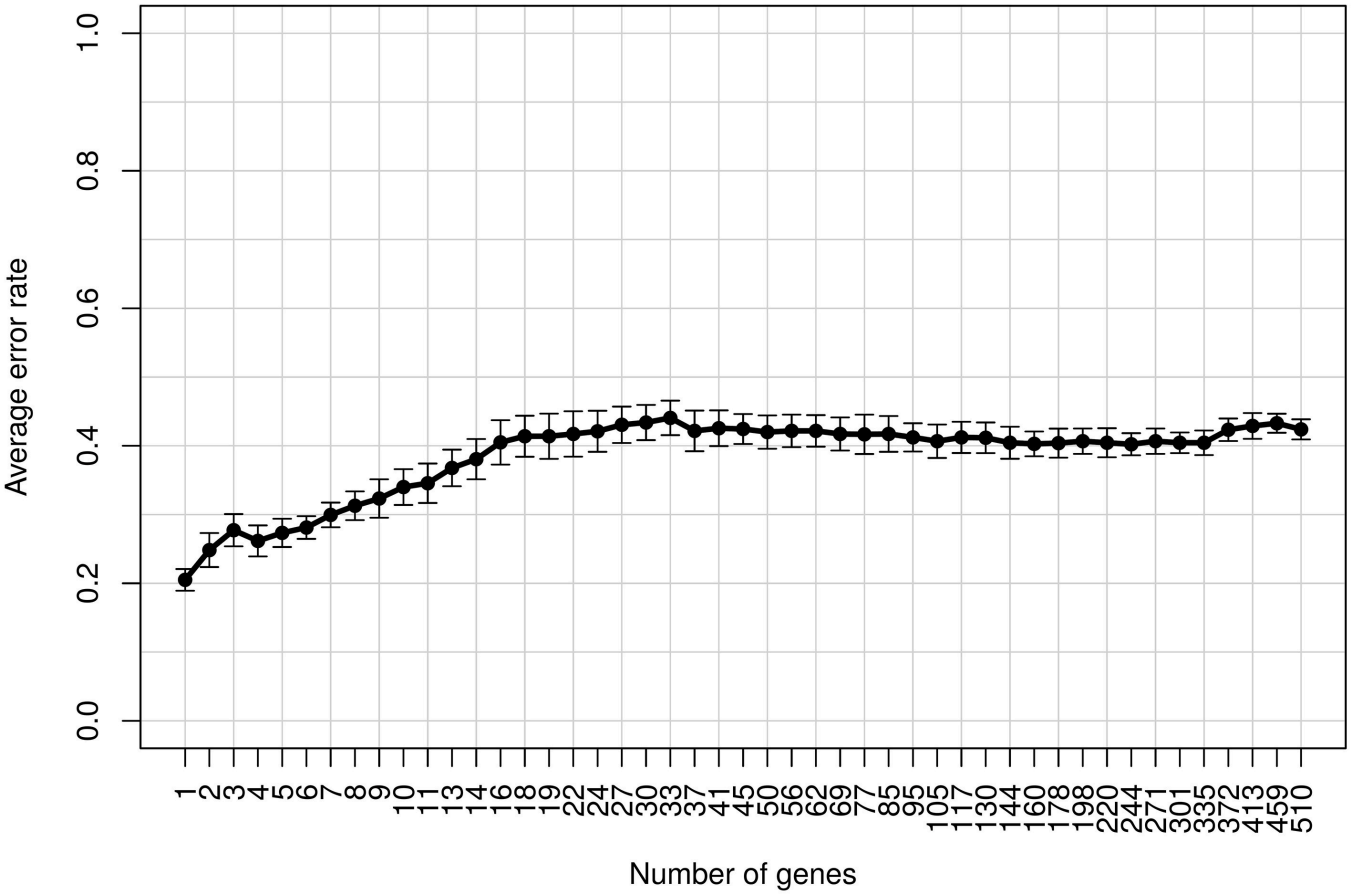
**The average error rates and standard deviations across the decreasing number of genes in the feature selection process**. The smallest error rate was calculated for using one gene, *S100B*.

**Figure 4:**
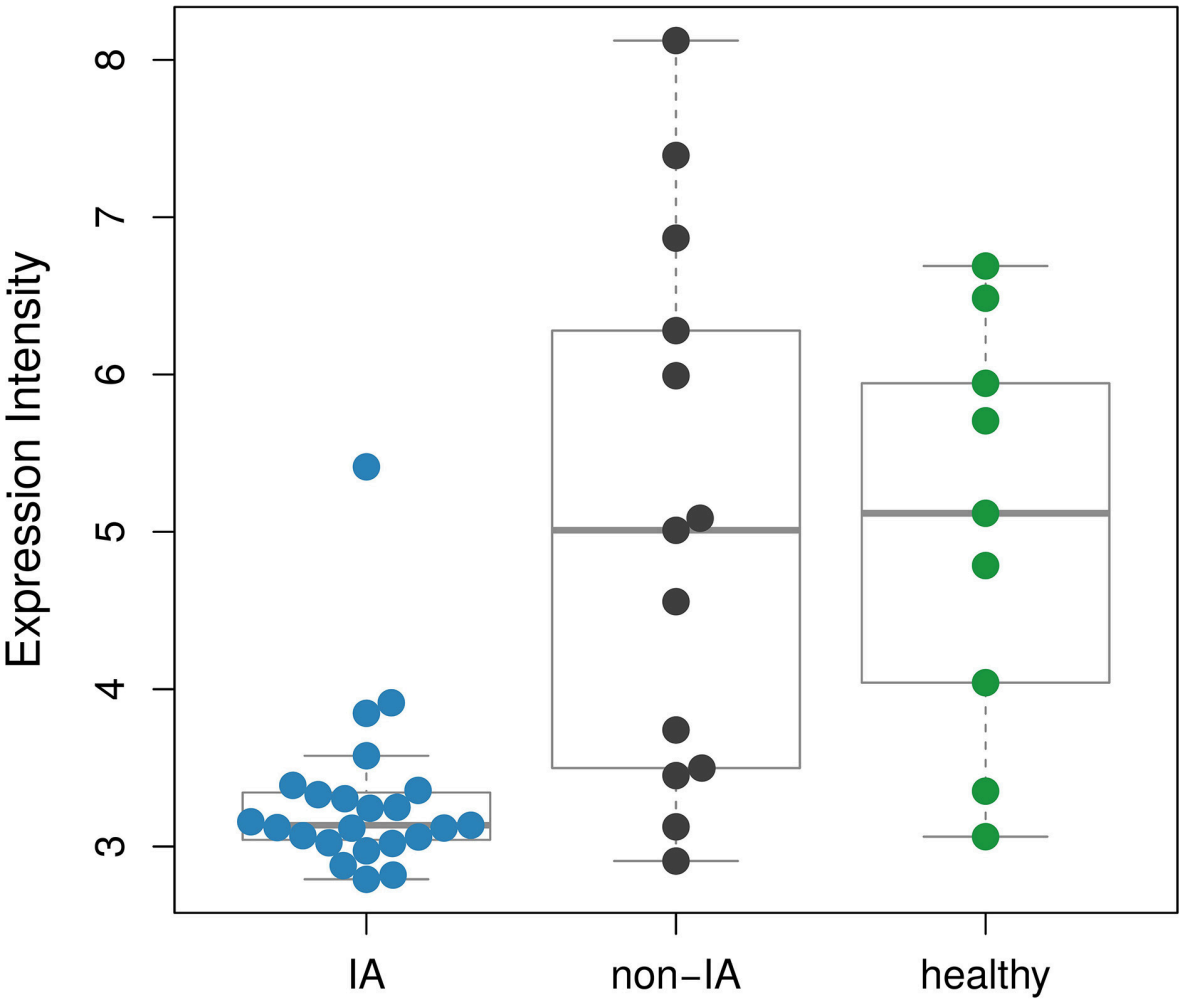
**Comparison of the expression intensities of ***S100B*** between the different conditions**. The distribution of the values of the non-IA samples covers a broad range and is similar to the healthy controls. The IA samples show low *S100B* expressions.

We aim to select biomarkers, which are specific for IA. For this purpose, we applied a feature selection. Briefly, feature selection is a technique that reduces the gene set to the most informative genes by removing irrelevant ones. In particular, we performed a recursive feature elimination (RFE) with random forest to identify biomakers (see Section 2). RFE is an iterative algorithm, where the features of a dataset are ranked and the worst scoring features are discarded in a stepwise manner. In each step, an error rate is computed using a classification algorithm. This error rate represents the proportion of wrong classifications. The feature subset yielding the smallest error rate is then selected. We used the DEGs of IA and non-IA patients as input for the RFE, which was wrapped in a repeated leave-one-out cross-validation. We discarded the worst scoring 10% of the features in each RFE step. The minimum error rate of 20.5% was calculated for using only a single transcript (Figure 3), which is *S100B*. The class-wise error rates are 17.7% for IA and 25.4% for non-IA patients (Table 2). According to this classification result, IA can be identified with a sensitivity of 82.3% and a specificity of 74.7%. When examining the expression of *S100B* across the different conditions (Figure 4), we found that it covers a broad range of intensity for samples collected from healthy individuals and non-IA patients. In contrast, in IA samples, the majority of the expression values are close to the lower bound.

**Table 2:** **Confusion table of the best feature selection result, where only ***S100B*** was used for classification**.

		**Predicted class**		
		**IA**	**Non-IA**	**Error rate %**
True class	IA	18.92	4.08	17.74
	Non-IA	3.30	9.70	25.38

### 3.3. Experimental validation of *S100B, MAOA*, and *SEMA4A* gene expression

To validate the gene expression levels obtained by microarray analysis, we performed real-time reverse transcription-PCR (RT-PCR) assays. Therefore, cDNA was reverse-transcribed from the same RNA samples used for hybridization onto the microarrays. Expression levels of *S100B, MAOA*, and *SEMA4A* were calculated relative to average IA levels. Expression patterns were similar comparing the microarray and the real-time RT-PCR analysis. Therefore, we could confirm the diminished expression of all three genes in PBMCs derived from patients suffering from IA compared to non-IA patients and healthy individuals.

### 3.4. S100B quantification by ELISA assay

In total, we collected 127 sera (among them, *n* = 75 sera from patients with IA, *n* = 52 sera from hematological patients without IA). In 91 of the 127 samples, S100B levels were below the detection limit of the assay. However, in sera collected in parallel or subsequently to a positive GM ELISA result (in patients suffering from proven or probable IA), concentrations of S100B were markedly lower (mean 5.81 pg/ml, range 2.8–19.5 pg/ml), compared to serum levels of hematological patients without IA (mean 32.0 pg/ml, range 15.4–45.8 pg/ml). This confirms the observation from the gene expression profiles, where patients with IA showed markedly reduced expression of *S100B* at the diagnosis of IA and in subsequent specimens. Therefore, *S100B* may serve as an additional biomarker for IA, complementary to the established methods.

### 3.5. SNP analyses in *S100B* and *AGER*

Genotyping of rs9722 [*S100B*], rs2070600 [*AGER*], and rs1800624 [*AGER*] revealed a significant increased susceptibility to IA if the polymorphism rs2070600 (G82S, GG/AG, *p* = 0.018) is present in patients after alloSCT (Table 3). In contrast, rs9722 and rs1800624 did not predispose to IA (*p* = 0.489 and *p* = 0.1554, respectively). These results confirm the observations of Cunha et al. (2011), who reported the association of SNPs in the S100B/RAGE axis with IA. In addition, rs9722 has previously been described to underlie increased serum levels of S100B in healthy individuals (Hohoff et al., 2010). Furthermore, Miller et al. (2013) were able to show that rs2070600 determines RAGE levels in the serum of patients with chronic obstructive pulmonary disease (COPD). Taking together, our data underline the prominent role of genetic markers in the S100B/RAGE axis and their potential relevance in controlling IA.

**Table 3:** **Genotype distributions of ***RAGE*** and ***S100B*** polymorphisms in recipients of stem cell transplants affected by IA and controls**.

**Genotype**	**Case (probable IA) (*n* = 33)**	**Control (*n* = 38)**	***p*-value**
wt AGER 1	28 (84.8%)	38 (100 %)	
SNP AGER 1	5 (15.2%)	0 (0 %)	0.0182
wt AGER 2	20 (60.6%)	16 (42.1 %)	
SNP AGER 2	13 (39.4%)	22 (57.9 %)	0.1554
wt S100B	30 (90.9%)	32 (84.2%)	
SNP S100B	3 (9.1%)	6 (15.8%)	0.489

*wt, wildtype; SNP, single nucleotide polymorphism. wt AGER 1 (rs2070600), GG genotype; SNP AGER (rs2070600), GA + AA genotypes; wt AGER 2 (rs1800624), TT genotype; SNP AGER (rs1800624), TA + AA genotypes; wt S100B (rs9722), CC genotype; SNP S100B (rs9722), CT + TT genotypes. P-values were calculated with the Fisher's exact test*.

### 3.6. *S100B* is differentially regulated by *A. fumigatus*

**Figure 5:**
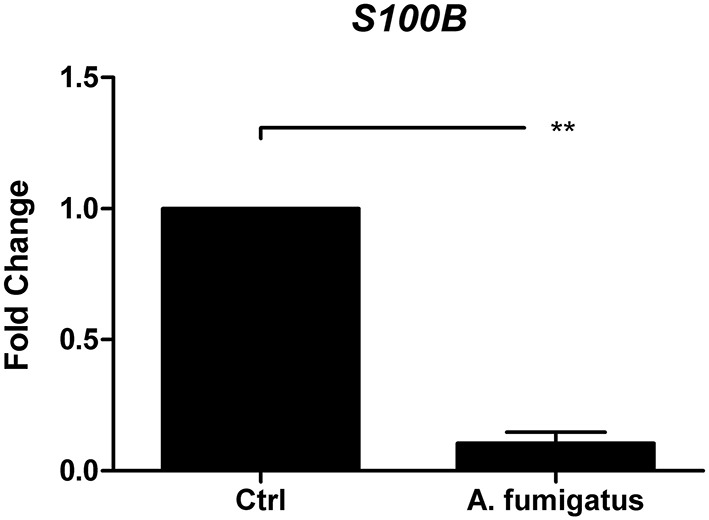
*****In vitro*** analysis of ***S100B*** regulation in DCs**. S100B was down-regulated in *A. fumigatus* stimulated DCs. DCs were either stimulated with *A. fumigatus* (MOI 1) or left untreated. mRNA level were quantified after 6 h by real-time PCR relative to reference gene *ALAS1*. Data of three independent experiments is illustrated as mean plus SEM (^**^p < 0.05 Student's paired *t*-test).

Dendritic cells (DCs) play an important role in pathogen recognition. DCs recognize pathogens via PRRs and bridge the innate and adaptive immune system (Wüthrich et al., 2012). The S100B gene regulation was examined *in vitro* after 12 h co-cultivation of DCs with inactivated *A. fumigatus* germ tubes. An *A. fumigatus* dependent reduction of S100B on gene expression level was confirmed (Figure 5).

### 3.7. Analysis of *S100B* gene knockdown on downstream cytokine levels

**Figure 6:**
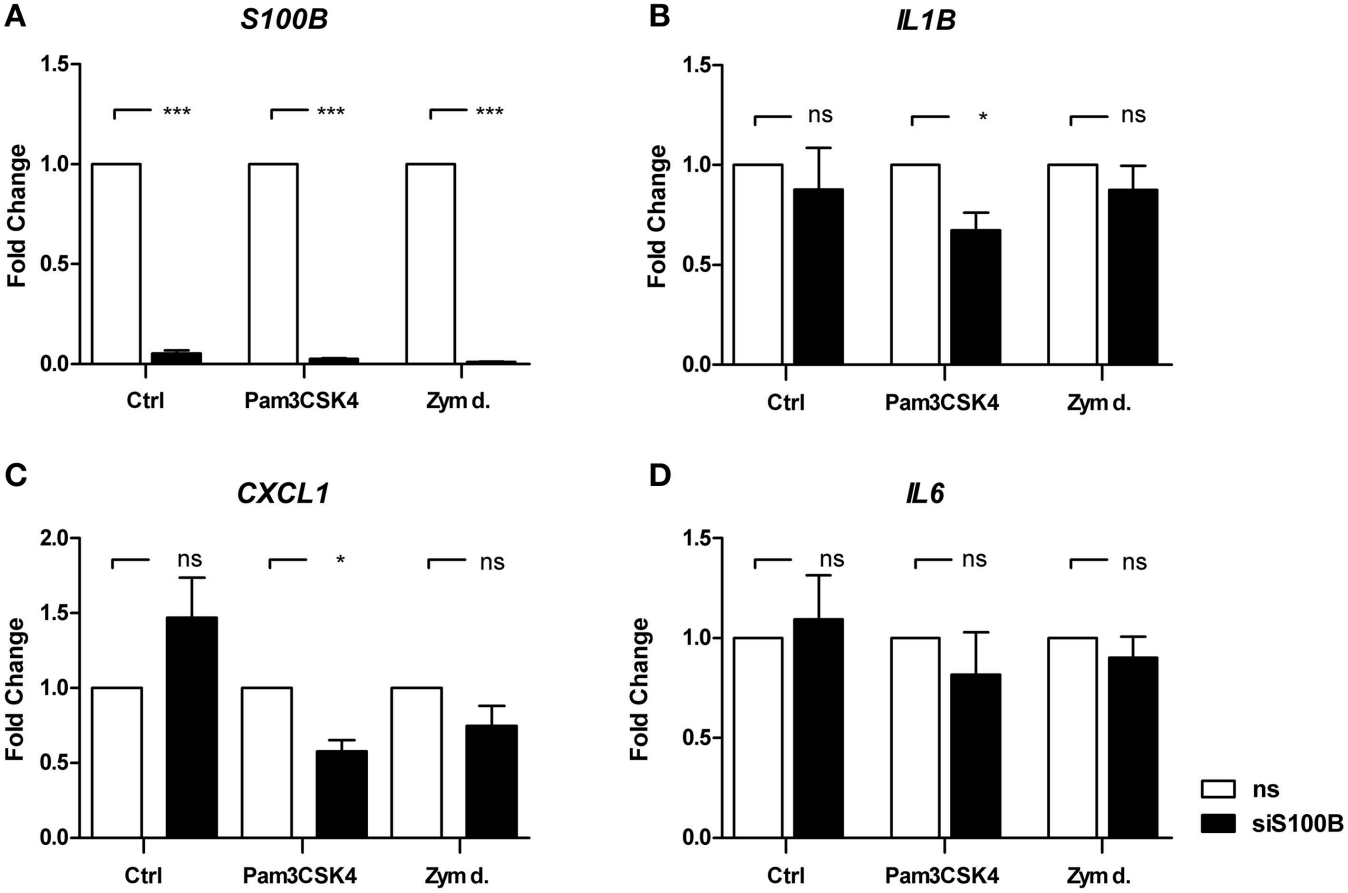
**Influence of ***S100B*** knockdown on gene regulation**. DCs were transfected by electroporation with either non-silencing siRNA (white bars) or with siRNA targeting *siS100B* (black bars). Twenty-four hours after electroporation, DCs were stimulated with zymosan depleted (100 μg/ml), Pam3CSK4 (100 ng/ml) (Invivogen), or left untreated. mRNA levels of *S100B* **(A)**, *IL1B* **(B)**, *CXCL1* **(C)**, and *IL6* **(D)** were quantified after 6 h by real-time PCR relative to non-silencing control. *ALAS1* served as reference gene. Data of four independent experiments is illustrated as mean plus SEM (^*^p < 0.01, ^***^p < 0.001 Student's paired *t*-test).

To further examine the relevance of S100B in *A. fumigatus* infection, we analyzed its role in the regulation of inflammatory cytokine responses. Thus, we stimulated DCs with defined PRR ligands activating TRL2/TRL1 or Dectin-1. Both, Dectin-1 and TLR2 are involved in *A. fumigatus* recognition of DCs. The synthetic triacylated lipoprotein Pam3CSK4 was used for the activation of TLR2/TLR1 signaling and depleted zymosan, which is a β-glucan, for Dectin-1 activation. We selected *IL1B, CXCL1*, and *IL6* and transfected DCs with siRNA targeting *S100B*. For these experiments, DCs were electroporated with *S100B* siRNA and random, non-silencing (ns) siRNA treated cells served as controls (Figure 6). siRNA transfection resulted in a > 90% *S100B* transcript reduction compared to the non-silencing control (Figure 6A). No influence of *S100B* knockdown was observed on *IL6* gene expression (Figure 6D). Upon stimulation with depleted zymosan, only a weak and insignificant reduction was observed. However, the *S100B* knockdown led to a significantly reduced expression of *IL1B* and *CXCL1* if DCs were activated with Pam3CSK4 (Figures 6B,C).

## 4. Discussion

In this study, we investigated the regulatory differences between IA patients and non-IA patients on the genome level and beyond. As a first step, we performed a GO analysis to reveal functional relations between DEGs. The analysis yielded multiple significantly over-represented GO categories connected to T cell activation and differentiation for IA-specific DEGs. This observation reflects the important role of T cells and thus the adaptive immune response in antifungal immune defense. In patients undergoing allogeneic SCT, both neutrophils and macrophages reconstitute relatively early. Nevertheless, *A. fumigatus* infections occur also after this recovery, indicating that the adaptive immune system contributes significantly to the control of *A. fumigatus* as well. One potential explanation for this observation is the prolonged immunosuppression conducted for the prevention and treatment of graft-versus-host disease (GvHD) (Cenci et al., 1997). This hypothesis is strengthened by numerous data from murine models showing that a previous infection with sublethal doses of *A. fumigatus* conidia or other fungal antigens protects mice against lethal re-challenge with the pathogen. Furthermore, adoptive transfer of CD4^+^ T cells from immunized animals transfers protective immunity to otherwise susceptible naïve recipient mice, stressing again the crucial role of adaptive immunity in protecting the host (Cenci et al., 2000). In addition to these data, murine experiments applying conditions favoring either a T_*H*_1 or T_*H*_2 CD4^+^ T cell response revealed that only T_*H*_1 CD4^+^ T cells protected the host from lethal challenge with *A. fumigatus*. By contrast, the induction of T_*H*_2 responses often even exacerbated disease. Furthermore, experimental data from mice infected with *A. fumigatus* implicate that not only the balance between T_*H*_1 cell and T_*H*_2 cell is important in controlling *A. fumigatus* infections but that also T_*reg*_ prevent excessive immune reaction in mice infected with *A. fumigatus* (Ito et al., 2006).

We used gene expression data from microarrays for the identification of biomarkers. Microarrays naturally produce data of high dimensionality. This data allows us to examine a broad range of genes for potential transcriptomic biomarkers for IA. The identification of biomarker genes for IA requires the reduction of the gene set to those genes which exhibit a distinct expression signature compared to non-IA patients. The process of dimension reduction by determining the most relevant genes and removing the non-informative ones is called feature selection. Feature selection methods are typically categorized into three types: filter, wrapper, and embedded techniques (Saeys et al., 2007). In this study, we used the wrapper approach by performing a RFE with random forest. Random forest is an effective classification algorithm that has shown good performance in a wide range of biomarker identification studies (Yan et al., 2012; Dix et al., 2015; Tremoulet et al., 2015). According to Ambroise and McLachlan (2002), it is important that all feature selection steps are performed within an outer cross-validation loop. Otherwise, a selection bias is introduced as the samples that are tested in each step already would contain some information about the differences between the classes. Thus, the test samples would not be independent. We meet this requirement by conducting the leave-one-out cross-validation as the outer loop of the feature selection process. Prior to the feature selection, we determined differentially expressed genes. However, this is not part of the feature selection, because we did not analyze for differences between the IA and the non-IA class. Instead, we determined the DEGs in comparison to the healthy control samples. In this way, we ensure that the IA biomarkers are connected to the therapy and thus also distinguish the patients from healthy blood donors.

*S100B* was identified as the most relevant gene for distinguishing between IA and non-IA samples. The calcium-binding protein S100B belongs to the damage-associated molecular patterns (DAMPs), which alert the immune system to the presence of tissue damage. Together with pathogen-associated molecular patterns (PAMPs), DAMPs play a major role in regulating the inflammatory response to pathogens. It is well known that progressive inflammation worsens disease and even impedes pathogen eradication. In consequence, fine tuning of the immune response and dispensing inflammation and pathogen elimination by leveling PAMP and DAMP driven responses is an ultimate prerequisite for a successful immune response against *A. fumigatus*. Thereby, S100B plays a crucial role. Thereon, upon intracellular binding of S100B to nucleic acids, it activates a TLR3/TLR9/TRIF-dependent pathway, culminating finally in the transcriptional down-regulation of *S100B*. These authors conclude that this spatiotemporal role provides evidence for S100B to be a central regulator of inflammation and pathogen sensing.

RNA from patients suffering from probable IA (at the time of a positive GM ELISA assay and following specimens) showed very low or absent expression of *S100B* while patients without any clinical signs of IA and healthy control persons showed variable *S100B* expression levels. Little is known about the regulatory mechanisms of *S100B* transcription. Samples analyzed in our study were taken relatively late in the course of IA as the detection of galactomannan in peripheral blood was a prerequisite for blood sampling. In consequence, macrophages, neutrophils, and dendritic cells were already activated and cytokines and reactive oxygen species were released. However, permanent activation of TLR2 and its downstream pro-inflammatory pathways promotes adverse effects with uncontrolled cytokine release and tissue damage. Thus, inhibited expression of *S100B* in patients with IA may reflect a self-protecting consequence of preceding TLR2 activation on PMN and other phagocytes and may at this later stage help to prevent uncontrolled and chronic inflammation and subsequent lung tissue damage.

Cunha et al. (2011) were able to demonstrate that human PBMC, stimulated for 2 h with *A. fumigatus* conidia or zymosan *in vitro* showed enhanced *S100B* levels compared to unstimulated control cells. However, our study revealed that stimulation of human monocyte-derived DCs for 12 h with *A. fumigatus* germ tubes led to significantly decreased *S100B* levels, concluding that at these later time points, S100B displays regulatory characteristics.

To further shed light on the potential relevance of S100B in the immune defense against *A. fumigatus*, functional *in vitro* studies are useful. The transfection of cells with specific siRNA is a common procedure to knockdown defined genes, usually followed by the subsequent characterization of specific downstream effectors (Sioud, 2015). To assess the function of S100B in the inflammatory response against *A. fumigatus*, an *S100B* knockdown was established in DCs. This knockdown was highly significant. To examine the role of *S100B*, an early time point was chosen, when *S100B* was not already affected by *A. fumigatus* stimulation. After 6 h of co-culturing, *S100B* was not affected by *A. fumigatus* in the non-silenced cells (not shown). Interestingly, we saw a significant impact of *S100B* knockdown on *IL1B* and *CXCL1* gene expression in Pam3CSK4 activated cells, whereas only a weak and not significant influence on Dectin-1 activation by depleted zymosan was observed. A correlation of S100B and IL1B induction via Sp1 and NF-κB was already described for primary microglia cells (Liu et al., 2005). Furthermore, in microglia, S100B is down-regulated by IFNγ and it was shown to relocate around phagosomes during *C. neoformans* infections (Adami et al., 2001). Since DCs are capable of IFNγ production, we hypothesize that an autocrine regulation mechanism might be possible. Moreover, TLR2 specificity by Pam3CSK4 activation was confirmed (Sorci et al., 2011). However, and in contrast to these authors, no induction of *S100B* was observed after 12 h co-cultivation, possibly due to different infection models.

Genotyping of rs9722 [*S100B*], rs2070600 [*AGER*], and rs1800624 [*AGER*] revealed a significantly increased susceptibility to IA if the polymorphism rs2070600 (G82S, GG/AG, *p* = 0.018) is present in patients after alloSCT (Table 3). In contrast, rs9722 and rs1800624 did not predispose to IA (*p* = 0.489 and *p* = 0.1554, respectively). These results confirm the observations of Cunha et al. (2011), who reported the association of SNPs in the S100B/Rage axis with IA. In addition, rs9722 has previously been described to underlie increased serum levels of S100B in healthy individuals (Hohoff et al., 2010). Furthermore, Miller et al. (2013) were able to show that rs2070600 determines RAGE levels in the serum of COPD patients. Taking together, our data underline the prominent role of genetic markers in the S100B/RAGE axis and their potential relevance in controlling IA.

Our results base on a limited set of data, patient and sample numbers. In addition, hematological patients, including patients after alloSCT are multi-morbid and suffer from a large variety of different complications, including graft-versus-host disease, relapse and a broad range of infections, especially caused by viruses. In consequence, data interpretation is difficult and very often effects localized upstream or downstream of the respective gene are evened because innate immunity pathways, such as the Toll like—MyD88—NF-κB/TRIF pathways are highly conserved and redundant. Therefore, validation studies with larger sample sizes are mandatory. In addition, it might be relevant to quantify levels of *S100B* in samples collected prior to the diagnosis of IA, e.g., by prospective sampling after alloSCT. Furthermore, *S100B* quantification in patients suffering from severe bacterial infections involving TLR2 activation might be relevant. However, with this pilot study, it became conceivable that S100B may serve as an additional human biomarker for IA and may upgrade the value of already well established fungal biomarkers. Furthermore, transcriptional profiling provides new findings on the immunopathology of IA and on the response of immunocompromised patients to *A. fumigatus*. This data help to better understand this devastating disease and to develop new targeted diagnostic and therapeutic options.

## Ethics statement

This study was approved by the Ethical Committee of the University Hospital of Wuerzburg (Approval 173/11: Invasive aspergillosis: Biomarkers for prevention, diagnosis and treatment response).

## Author contributions

AD did the bioinformatic analysis and co-wrote the manuscript. JS managed clinical data and co-wrote the manuscript. KC, MF, and AS performed the validation experiments, including the RNAi knockdown and co-wrote the manuscript. MB generated the microarray data and co-wrote the manuscript. RG, JLi, HE, and JLö designed the study and co-wrote the manuscript.

### Conflict of interest statement

The authors declare that the research was conducted in the absence of any commercial or financial relationships that could be construed as a potential conflict of interest.

## Footnotes

^1^http://brainarray.mbni.med.umich.edu/Brainarray/Database/CustomCDF/19.0.0/entrezg.asp.

^2^http://biomart.genenames.org/.
