# Supplementary material for: Genome-Wide Expression Profiling Reveals S100B as Biomarker for Invasive Aspergillosis
Source: Front Microbiol. 2016 Mar 21;7:320. doi: 10.3389/fmicb.2016.00320 (PMC4800190; doi:10.3389/fmicb.2016.00320)
Supplement: Supplementary file 1 [file DataSheet1.PDF]

Supplementary Material: Genome-wide Expression Profiling in Haematological Patients Suffering from Invasive Aspergillosis

Andreas Dix, Kristin Czakai, Jan Springer, Mirjam Fliesser, Michael Bonin, Reinhard Guthke, Anna Lena Schmitt, Hermann Einsele, Jörg Linde *, and Jürgen Löffler *

*Correspondence:
Jörg Linde
joerg.linde@leibniz-hki.de

Jürgen Löffler
University Hospital Würzburg, Medical Hospital II, Würzburg, Josef-Schneider-Str. 2, 97080, Germany, Loeffler_J@ukw.de

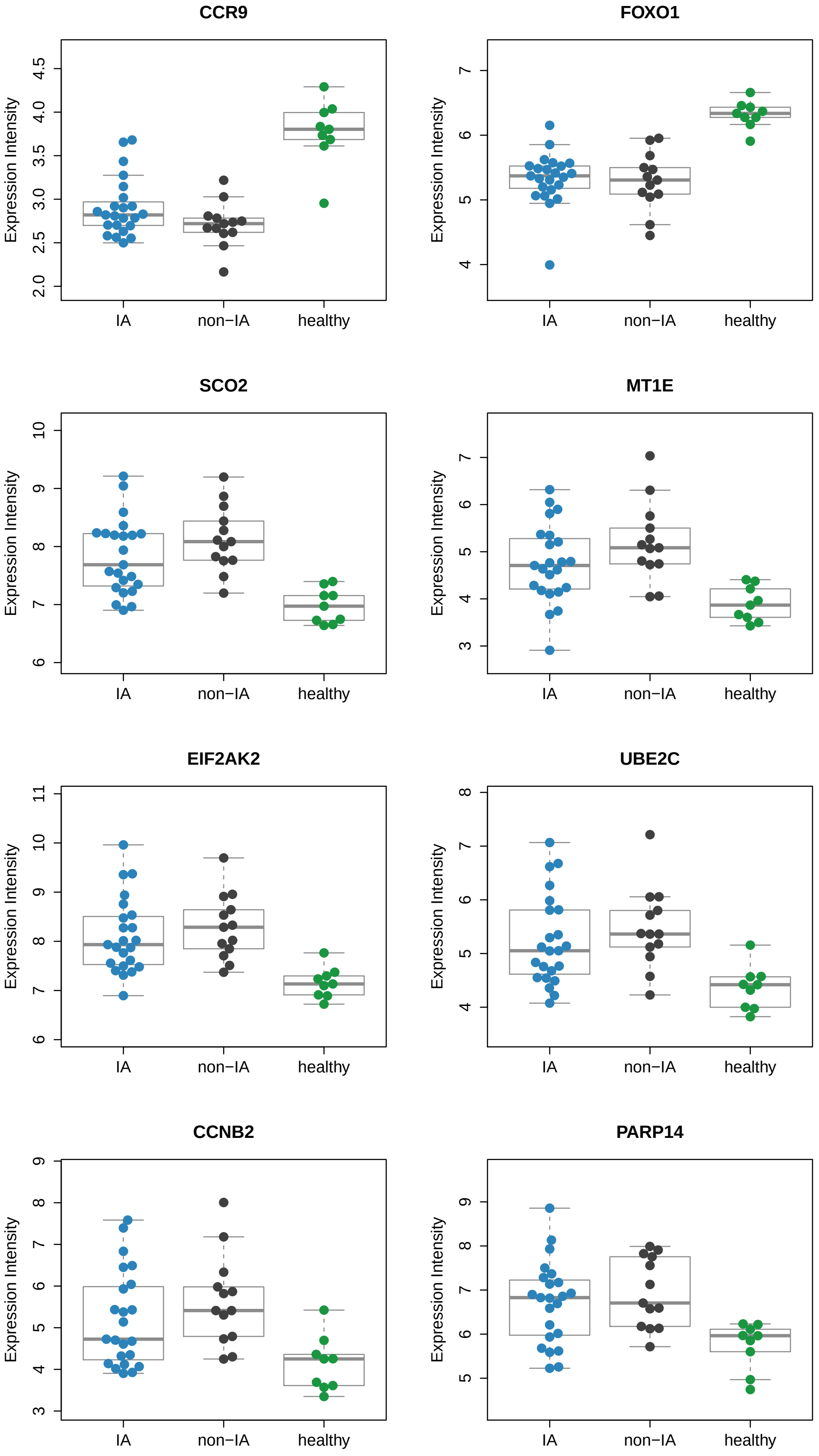

Supplementary Figure 1. The gene expression intensities of the 8 DEGs which are specific for the non-IA samples. The differences between the IA and the non-IA samples are very small.

Supplementary Table 1. The list of over-represented Gene Ontology terms based on the differentially expressed genes which are common for the IA and non-IA samples. The table was generated using GOrilla and only terms with a FDR-adjusted significance value of at least 0.05 were considered.

GO Term	Description	P-value	FDR q-value
GO:0006959	humoral immune response	1.56E-11	2.14E-07
GO:0050853	B cell receptor signaling pathway	8.36E-11	3.84E-07
GO:0006958	complement activation, classical pathway	8.36E-11	5.75E-07
GO:0002376	immune system process	6.71E-10	2.31E-06
GO:0006956	complement activation	1.00E-09	2.68E-06
GO:0002682	regulation of immune system process	4.20E-09	9.64E-06
GO:0002684	positive regulation of immune system process	6.17E-09	1.21E-05
GO:0072376	protein activation cascade	2.16E-08	3.71E-05
GO:0006955	immune response	8.64E-08	1.32E-04
GO:0002252	immune effector process	1.39E-07	1.92E-04
GO:0042113	B cell activation	1.73E-07	2.16E-04
GO:0007166	cell surface receptor signaling pathway	3.12E-07	3.31E-04
GO:0002250	adaptive immune response	2.99E-07	3.43E-04
GO:0048583	regulation of response to stimulus	6.21E-07	5.70E-04
GO:0050864	regulation of B cell activation	6.06E-07	5.97E-04
GO:0002253	activation of immune response	1.02E-06	8.74E-04
GO:0050776	regulation of immune response	2.55E-06	2.06E-03
GO:0042100	B cell proliferation	2.98E-06	2.28E-03
GO:0050778	positive regulation of immune response	5.32E-06	3.85E-03
GO:0050789	regulation of biological process	7.12E-06	4.91E-03
GO:0006952	defense response	8.89E-06	5.56E-03
GO:0006910	phagocytosis, recognition	8.60E-06	5.64E-03
GO:0006911	phagocytosis, engulfment	1.29E-05	7.70E-03
GO:0002429	immune response-activating cell surface receptor signaling pathway	1.69E-05	9.32E-03
GO:0065007	biological regulation	1.67E-05	9.57E-03
GO:0030183	B cell differentiation	1.86E-05	9.84E-03
GO:0050851	antigen receptor-mediated signaling pathway	2.00E-05	1.02E-02
GO:0030098	lymphocyte differentiation	2.90E-05	1.42E-02
GO:0001775	cell activation	4.04E-05	1.92E-02
GO:0046649	lymphocyte activation	4.43E-05	2.03E-02
GO:0010324	membrane invagination	4.62E-05	2.05E-02
GO:0045321	leukocyte activation	4.90E-05	2.05E-02
GO:0050896	response to stimulus	4.84E-05	2.08E-02
GO:0002757	immune response-activating signal transduction	5.26E-05	2.13E-02
GO:0002768	immune response-regulating cell surface receptor signaling pathway	5.79E-05	2.22E-02
GO:0002521	leukocyte differentiation	5.74E-05	2.26E-02
GO:0002764	immune response-regulating signaling pathway	8.51E-05	3.17E-02
GO:0050871	positive regulation of B cell activation	1.07E-04	3.88E-02
GO:0002694	regulation of leukocyte activation	1.15E-04	4.06E-02

Supplementary Table 2. The list of over-represented Gene Ontology terms based on the differentially expressed genes which are specific for the IA samples. The table was generated using GOrilla and only terms with a FDR-adjusted significance value of at least 0.05 were considered.

GO Term	Description	P-value	FDR q-value
GO:0002376	immune system process	6.03E-21	8.30E-17
GO:0006955	immune response	2.89E-13	2.00E-09
GO:0002684	positive regulation of immune system process	2.63E-12	9.07E-09
GO:0002682	regulation of immune system process	2.58E-12	1.18E-08
GO:0042110	T cell activation	5.35E-11	9.21E-08
GO:0071593	lymphocyte aggregation	6.55E-11	1.00E-07
GO:0070489	T cell aggregation	5.35E-11	1.05E-07
GO:0030217	T cell differentiation	4.72E-11	1.08E-07
GO:0002694	regulation of leukocyte activation	8.77E-11	1.21E-07
GO:0006952	defense response	4.68E-11	1.29E-07
GO:0070486	leukocyte aggregation	1.18E-10	1.48E-07
GO:0045321	leukocyte activation	1.48E-10	1.70E-07
GO:0050776	regulation of immune response	2.18E-10	2.31E-07
GO:0050865	regulation of cell activation	5.65E-10	5.56E-07
GO:0006614	SRP-dependent cotranslational protein targeting to membrane	7.48E-10	6.87E-07
GO:0050778	positive regulation of immune response	1.12E-09	9.09E-07
GO:0006613	cotranslational protein targeting to membrane	1.07E-09	9.18E-07
GO:0046632	alpha-beta T cell differentiation	1.39E-09	1.00E-06
GO:0045047	protein targeting to ER	1.50E-09	1.04E-06
GO:0034109	homotypic cell-cell adhesion	1.38E-09	1.05E-06
GO:0007159	leukocyte cell-cell adhesion	1.85E-09	1.21E-06
GO:0051251	positive regulation of lymphocyte activation	2.36E-09	1.47E-06
GO:0046649	lymphocyte activation	2.62E-09	1.57E-06
GO:0072599	establishment of protein localization to endoplasmic reticulum	2.90E-09	1.67E-06
GO:0002696	positive regulation of leukocyte activation	3.43E-09	1.89E-06
GO:0050867	positive regulation of cell activation	6.58E-09	3.24E-06
GO:0002521	leukocyte differentiation	6.42E-09	3.28E-06
GO:0070972	protein localization to endoplasmic reticulum	6.27E-09	3.32E-06
GO:0002253	activation of immune response	1.00E-08	4.76E-06
GO:0046631	alpha-beta T cell activation	1.34E-08	6.13E-06
GO:0019083	viral transcription	1.49E-08	6.60E-06
GO:0000184	nuclear-transcribed mRNA catabolic process, nonsense-mediated decay	3.61E-08	1.55E-05
GO:0001775	cell activation	4.36E-08	1.82E-05
GO:0006954	inflammatory response	5.21E-08	2.11E-05
GO:0030098	lymphocyte differentiation	5.74E-08	2.26E-05
GO:0051249	regulation of lymphocyte activation	6.40E-08	2.45E-05
GO:0016337	single organismal cell-cell adhesion	7.45E-08	2.63E-05
GO:0050870	positive regulation of T cell activation	7.40E-08	2.68E-05
GO:0045087	innate immune response	7.31E-08	2.72E-05
GO:0034112	positive regulation of homotypic cell-cell adhesion	1.03E-07	3.55E-05
GO:1903039	positive regulation of leukocyte cell-cell adhesion	1.12E-07	3.66E-05
GO:0031295	T cell costimulation	1.10E-07	3.70E-05
GO:0031294	lymphocyte costimulation	1.30E-07	3.97E-05
GO:0001817	regulation of cytokine production	1.29E-07	4.05E-05

GO:0000956	nuclear-transcribed mRNA catabolic process	1.29E-07	4.13E-05
GO:0002757	immune response-activating signal transduction	1.95E-07	5.85E-05
GO:0098602	single organism cell adhesion	2.28E-07	6.69E-05
GO:0043604	amide biosynthetic process	2.70E-07	7.75E-05
GO:0022409	positive regulation of cell-cell adhesion	2.91E-07	8.19E-05
GO:0006402	mRNA catabolic process	3.74E-07	1.03E-04
GO:0045785	positive regulation of cell adhesion	5.36E-07	1.45E-04
GO:0006401	RNA catabolic process	6.63E-07	1.75E-04
GO:0080134	regulation of response to stress	7.78E-07	2.02E-04
GO:0051704	multi-organism process	7.95E-07	2.03E-04
GO:0019439	aromatic compound catabolic process	8.44E-07	2.11E-04
GO:0002764	immune response-regulating signaling pathway	9.04E-07	2.22E-04
GO:0019058	viral life cycle	1.01E-06	2.44E-04
GO:0006414	translational elongation	1.03E-06	2.46E-04
GO:0001819	positive regulation of cytokine production	1.08E-06	2.52E-04
GO:0050863	regulation of T cell activation	1.12E-06	2.57E-04
GO:0043043	peptide biosynthetic process	1.26E-06	2.84E-04
GO:0050852	T cell receptor signaling pathway	1.59E-06	3.53E-04
GO:1903037	regulation of leukocyte cell-cell adhesion	1.77E-06	3.76E-04
GO:0048583	regulation of response to stimulus	1.75E-06	3.82E-04
GO:0006412	translation	1.77E-06	3.82E-04
GO:0031347	regulation of defense response	1.93E-06	4.03E-04
GO:0009607	response to biotic stimulus	1.97E-06	4.04E-04
GO:0048584	positive regulation of response to stimulus	2.00E-06	4.05E-04
GO:0006415	translational termination	2.05E-06	4.09E-04
GO:0043207	response to external biotic stimulus	2.12E-06	4.17E-04
GO:0022411	cellular component disassembly	2.63E-06	5.11E-04
GO:0034110	regulation of homotypic cell-cell adhesion	3.07E-06	5.88E-04
GO:0043241	protein complex disassembly	3.14E-06	5.92E-04
GO:1901361	organic cyclic compound catabolic process	3.28E-06	6.11E-04
GO:0034655	nucleobase-containing compound catabolic process	3.38E-06	6.21E-04
GO:0007155	cell adhesion	3.43E-06	6.21E-04
GO:0043624	cellular protein complex disassembly	3.59E-06	6.42E-04
GO:1903034	regulation of response to wounding	3.71E-06	6.47E-04
GO:0002252	immune effector process	3.86E-06	6.49E-04
GO:0006950	response to stress	3.69E-06	6.51E-04
GO:0022610	biological adhesion	3.81E-06	6.55E-04
GO:0050851	antigen receptor-mediated signaling pathway	3.86E-06	6.56E-04
GO:0090150	establishment of protein localization to membrane	4.00E-06	6.64E-04
GO:0006612	protein targeting to membrane	4.25E-06	6.97E-04
GO:0002292	T cell differentiation involved in immune response	4.95E-06	8.02E-04
GO:0032984	macromolecular complex disassembly	5.70E-06	9.13E-04
GO:0046700	heterocycle catabolic process	6.29E-06	9.96E-04
GO:0044270	cellular nitrogen compound catabolic process	6.58E-06	1.03E-03
GO:0050862	positive regulation of T cell receptor signaling pathway	6.68E-06	1.03E-03
GO:0098609	cell-cell adhesion	6.85E-06	1.05E-03
GO:0022407	regulation of cell-cell adhesion	8.24E-06	1.25E-03
GO:0050727	regulation of inflammatory response	8.33E-06	1.25E-03
GO:0043367	CD4-positive, alpha-beta T cell differentiation	8.61E-06	1.28E-03

GO:0002366	leukocyte activation involved in immune response	8.74E-06	1.28E-03
GO:0002263	cell activation involved in immune response	1.04E-05	1.50E-03
GO:0032496	response to lipopolysaccharide	1.21E-05	1.74E-03
GO:0002286	T cell activation involved in immune response	1.28E-05	1.82E-03
GO:1901564	organonitrogen compound metabolic process	1.36E-05	1.91E-03
GO:0006413	translational initiation	1.39E-05	1.93E-03
GO:0042093	T-helper cell differentiation	1.52E-05	2.07E-03
GO:0002294	CD4-positive, alpha-beta T cell differentiation involved in immune response	1.52E-05	2.09E-03
GO:0035710	CD4-positive, alpha-beta T cell activation	1.79E-05	2.42E-03
GO:0051707	response to other organism	2.06E-05	2.75E-03
GO:0002237	response to molecule of bacterial origin	2.22E-05	2.94E-03
GO:0034097	response to cytokine	2.36E-05	3.09E-03
GO:0002293	alpha-beta T cell differentiation involved in immune response	2.88E-05	3.70E-03
GO:0002287	alpha-beta T cell activation involved in immune response	2.88E-05	3.74E-03
GO:0072657	protein localization to membrane	3.05E-05	3.89E-03
GO:0030155	regulation of cell adhesion	3.14E-05	3.97E-03
GO:0009605	response to external stimulus	3.58E-05	4.44E-03
GO:0051240	positive regulation of multicellular organismal process	3.57E-05	4.47E-03
GO:0070269	pyroptosis	3.75E-05	4.61E-03
GO:0045576	mast cell activation	3.84E-05	4.68E-03
GO:0032101	regulation of response to external stimulus	6.86E-05	8.29E-03
GO:0009056	catabolic process	7.13E-05	8.54E-03
GO:0032649	regulation of interferon-gamma production	7.25E-05	8.61E-03
GO:1901566	organonitrogen compound biosynthetic process	8.13E-05	9.57E-03
GO:0006518	peptide metabolic process	8.40E-05	9.80E-03
GO:0033089	positive regulation of T cell differentiation in thymus	8.68E-05	9.96E-03
GO:2000400	positive regulation of thymocyte aggregation	8.68E-05	1.00E-02
GO:0002429	immune response-activating cell surface receptor signaling pathway	9.14E-05	1.03E-02
GO:0043123	positive regulation of I-kappaB kinase/NF-kappaB signaling	9.12E-05	1.04E-02
GO:0002250	adaptive immune response	1.02E-04	1.15E-02
GO:0043122	regulation of I-kappaB kinase/NF-kappaB signaling	1.03E-04	1.15E-02
GO:0043603	cellular amide metabolic process	1.08E-04	1.19E-02
GO:0045730	respiratory burst	1.23E-04	1.35E-02
GO:0002274	myeloid leukocyte activation	1.36E-04	1.47E-02
GO:0072594	establishment of protein localization to organelle	1.47E-04	1.58E-02
GO:0050896	response to stimulus	1.59E-04	1.70E-02
GO:0031349	positive regulation of defense response	1.63E-04	1.73E-02
GO:0002181	cytoplasmic translation	1.70E-04	1.77E-02
GO:0098542	defense response to other organism	1.69E-04	1.78E-02
GO:1903708	positive regulation of hemopoiesis	1.73E-04	1.79E-02
GO:0007098	centrosome cycle	1.82E-04	1.87E-02
GO:0002526	acute inflammatory response	2.19E-04	2.23E-02
GO:0030154	cell differentiation	2.26E-04	2.29E-02
GO:0050857	positive regulation of antigen receptor-mediated signaling pathway	2.28E-04	2.29E-02
GO:0045058	T cell selection	2.32E-04	2.31E-02
GO:0006605	protein targeting	2.68E-04	2.64E-02
GO:0048518	positive regulation of biological process	2.68E-04	2.65E-02
GO:0070670	response to interleukin-4	2.77E-04	2.71E-02
GO:0044236	multicellular organismal metabolic process	3.23E-04	3.13E-02

GO:0042742	defense response to bacterium	3.28E-04	3.16E-02
GO:0002768	immune response-regulating cell surface receptor signaling pathway	3.30E-04	3.16E-02
GO:0048522	positive regulation of cellular process	3.35E-04	3.18E-02
GO:0033993	response to lipid	3.50E-04	3.31E-02
GO:0032963	collagen metabolic process	3.80E-04	3.53E-02
GO:0045621	positive regulation of lymphocyte differentiation	3.80E-04	3.56E-02
GO:1901575	organic substance catabolic process	3.94E-04	3.64E-02
GO:0010467	gene expression	4.16E-04	3.82E-02
GO:0045089	positive regulation of innate immune response	4.31E-04	3.94E-02
GO:0014005	microglia development	4.51E-04	4.03E-02
GO:0001865	NK T cell differentiation	4.51E-04	4.06E-02
GO:0032633	interleukin-4 production	4.51E-04	4.08E-02
GO:1902533	positive regulation of intracellular signal transduction	4.85E-04	4.29E-02
GO:0002285	lymphocyte activation involved in immune response	4.83E-04	4.30E-02
GO:0031348	negative regulation of defense response	5.22E-04	4.58E-02
GO:0019693	ribose phosphate metabolic process	5.30E-04	4.62E-02
GO:0009156	ribonucleoside monophosphate biosynthetic process	5.38E-04	4.66E-02
GO:0044259	multicellular organismal macromolecule metabolic process	5.65E-04	4.86E-02
GO:0032655	regulation of interleukin-12 production	5.72E-04	4.89E-02
